# Exposure of adult sea urchin *Strongylocentrotus intermedius* to stranded heavy fuel oil causes developmental toxicity on larval offspring

**DOI:** 10.7717/peerj.13298

**Published:** 2022-04-19

**Authors:** Xuanbo Wang, Xishan Li, Deqi Xiong, Hang Ren, Huishu Chen, Zhonglei Ju

**Affiliations:** College of Environmental Science and Engineering, Dalian Maritime University, Dalian, China

**Keywords:** Heavy fuel oil, Sea urchin, Toxicity effect, Offspring

## Abstract

Heavy fuel oil (HFO) spills pose serious threat to coastlines and sensitive resources. Stranded HFO that occurs along the coastline could cause long-term and massive damage to the marine environment and indirectly affect the survival of parental marine invertebrates. However, our understanding of the complex associations within invertebrates is primarily limited, particularly in terms of the toxicity effects on the offspring when parents are exposed to stranded HFO. Here, we investigated the persistent effects on the early development stage of the offspring following stranded HFO exposure on the sea urchin *Strongylocentrotus intermedius*. After 21 d exposure, sea urchins exhibited a significant decrease in the reproductive capacity; while the reactive oxygen species level, 3-nitrotyrosine protein level, protein carbonyl level, and heat shock proteins 70 expression in the gonadal tissues and gametes significantly increased as compared to the controls, indicating that HFO exposure could cause development toxicity on offspring in most traits of larval size. These results suggested that the stranded HFO exposure could increase oxidative stress of gonadal tissues, impair reproductive functions in parental sea urchins, and subsequently impact on development of their offspring. This study provides valuable information regarding the persistent toxicity effects on the offspring following stranded HFO exposure on sea urchins.

## Introduction

Around 380 million gallons of oil are released into the marine each year due to natural disasters and anthropogenic factors (oil spill from ships and oil platforms) (Naz et al., 2021; Pisano et al., 2016). Oil spills profoundly impact the coastal and marine environments, adversely affecting aquatic organisms (Ansell et al., 2001). Previous studies have shown that oil-derived hydrocarbons negatively impact aquatic organisms. For example, polar cod (*Boreogadus saida*) exposed to crude oil showed sublethal effects on the lipid composition of liver tissue (Vieweg et al., 2022). Crude oil exposure has been shown to cause growth inhibition and lipid allocation on polar cod embryonic (*Boreogadus saida*) (Laurel et al., 2019). Oil-derived hydrocarbons exposure of sea urchin (*Strongylocentrotus intermedius*) significantly increased lipid peroxidation level and caused enhanced oxidative damage (Wang et al., 2021). In addition, nearly 40% of the more than 400 ship-sourced oil spills over the past few decades involved heavy fuel oil (HFO) (Ansell et al., 2001). The tidal cycle causes the seawater to contact the HFO gravel, promoting the release of sustained polycyclic aromatic hydrocarbons (PAHs) into the seawater when HFO stranded to the shore (Carls et al., 2003). PAHs can change in marine environments, altering the physiology of aquatic organisms (Ma et al., 2020; Wang et al., 2018). Previous studies showed that HFO stranded on gravel increased rainbow trout embryos (*Oncorhynchus mykiss*) mortality and was higher than crude oil (Martin et al., 2014). To our knowledge, there is limited understanding on the impacts of stranded HFO on marine benthic invertebrates. Benthic invertebrates with limited mobility generally live on the benthic substrate and are more vulnerable to threats than mobile species. Therefore, exploring the toxic effects of stranded HFO on benthic invertebrates can provide a new basis for the ecological risk assessment of HFO leakage.

Marine invertebrates have a complex life history, including short-term embryonic, larval, and long-term adult stages. The performance in one life-history stage may positively or negatively affect the subsequent life-history stages, the so-called developmental domino phenomena (Byrne, 2012). Marine invertebrates with short generation cycles have a strong potential for evolutionary adaptation owing to the environmental stress experienced through several generations (Chen & Stillman, 2012; Ding et al., 2020). However, whether stranded HFO causes persistent effects on the offspring in marine invertebrates remains unclear.

The slow movements are relatively impacting the adult sea urchins, as they have a though small, choice of substrate, mainly during larval settlement. In addition, sea urchin has a short generation cycle and have strong evolutionary adaptation potential. Therefore, sea urchins are a valid model for the study of oil pollution, because adults live and feed in close contact with the coastal bottom, with the ability of remodeling it by grazing. Exposure of adult sea urchins to environmental stress caused changes in their behavior and physiology (Zhang et al., 2017; Chiarelli et al., 2014). Such changes include tube foot withdrawal, decreased adsorption capacity, and spines falling off (Axiak & Saliba, 1981; Bielmyer et al., 2005). Therefore, sea urchin is a suitable experimental organism for assessing the environmental impact and ecological risk related to kind of marine stressors, such as heavy metals (Chiarelli, Martino & Roccheri, 2019), PAHs (Lister, Lamare & Burritt, 2016), nano plastics (Manzo & Schiavo, 2022) and other marine pollutants (Martino et al., 2022; Bellas et al., 2022). In addition, most investigations have devoted to parental fish exposed to crude oil could have adverse effects on embryos (Bautista et al., 2021; Jasperse et al., 2019; Li et al., 2021). However, less is known about the parental exposure of sea urchins to oil pollutants may have a negative impact on offspring. Therefore, considering the ecological importance and susceptibility to oil pollution, the persistent effects on the early development stage of the offspring of sea urchin exposure to stranded HFO pollution need to be explored.

Oil pollution may also cause the overproduction of reactive oxygen species (ROS) and reactive nitrogen species (RNS) in marine invertebrate. Previous studies have found that marine mussel (*Mytilus galloprovincialis*) exposed to crude oil increased ROS production, induced actin cytoskeleton disruption (Katsumiti et al., 2019). Crude oil exposure caused a significant increase in the ROS on sea cucumber (*Apostichopus japonicus*), further resulting in increased oxidative damage *in vivo* (Li et al., 2020). It has been documented that the increase of ROS and RNS within sea urchins negatively affects the DNA, proteins, and membrane lipids (Duan et al., 2018a; Zhou et al., 2012). Organisms are subjected to harmful endogenous or exogenous stresses, resulting in the overproduction of ROS and RNS in the body, leading to oxidative stress (Di Meo et al., 2016). The overproduction of ROS, which are highly active intermediate products formed by molecular oxygen during reduction, will affect homeostasis and induce stress response (Dröge, 2002). RNS are involved in the nitrosative stress in animal cells and tissues. Overproduction of RNS can induce an imbalance of 3-nitrotyrosine protein (3-NTP) in the body (Castellano et al., 2018). The overproduction of NTP may be associated with aging and diseases in organisms (Johnstone et al., 2019). Therefore, we have speculated that HFO exposure may result in ROS and RNS overproduction that would induce oxidative stress, thereby affecting the gonadal function of sea urchins. The changes in the gonadal function of sea urchins may affect gamete quality and offspring development.

This study aimed to investigate the impact of long-term stranded HFO exposure on gonadal functions, ROS, 3-NTP and protein carbonyl (PC) levels, *hsp*70 gene expression in gonads and gametes in the sea urchin *Strongylocentrotus intermedius*. We also aimed to investigate: (1) whether the long-term exposure of paternal and maternal sea urchins to stranded HFO causes toxic effects to their offspring; (2) whether the fitness of sea urchin offspring shows paternal or maternal effects following parental exposure to stranded HFO. The present study provides new and comprehensive important information related to toxicity effects of HFO on marine invertebrate’s offspring.

## Materials and Methods

### HFO gravel column tanks preparation

The preparation of HFO gravel column tanks followed methods as described in previous studies (Duan et al., 2018a; Marty, Heintz & Hinton, 1997), with some modifications. “380# HFO” was poured in 8 kg of clean gravel to obtain oil loading of 9,600 μg oil/g gravel. The HFO gravel was manually shaken using a polyvinyl chloride mixer for 5 min, which made the HFO evenly distributed on the gravel. The HFO gravel was kept in a dark place for 24 h to allow time for a thin oil film to completely coat the gravel. Then, the HFO gravel was transferred into an appropriate polyvinyl chloride pipe with a diameter and height of 45 and 55 cm, respectively. We used natural seawater pumped at a rate of 100 mL/min to wash the columns from bottom to top at 16 °C for 24 h and used a tank to collect the exposure fluid from the outlet of the gravel column for sea urchin exposure experiments. The control gravel column was applied with no oil.

Samples of seawater from the columns were collected every 24 h for chemical analysis to determine concentrations of total petroleum hydrocarbons (TPH) and PAHs in the exposure solution, as described in the previous studies (Li et al., 2019; Duan et al., 2018a). Briefly, 200 mL solution samples were extracted with 20 mL *n*-hexane using a separating funnel. TPH concentrations were measured by ultraviolet spectrometry (Epoch 2; BioTek, Winooski, VT, USA) according to the specification for seawater analysis (GB 17378.4-2007) method with modifications. Moreover, 16 priority PAHs were also measured by gas chromatography-mass spectrometry (GC/MS) (HP6890 GC-5975 MSD; Agilent Technologies Inc., Santa Clara, CA, USA). The EPA 3510C liquid-liquid extraction method was used to pretreat the water samples. EPA 3630C silica gel cleaning method is used to clean the concentrate, and samples were measured by GC/MS. TPH concentrations were decreasing from 811.22 μg/L to 474.64 μg/L (Fig. S1). PAHs concentrations decreased from 6.04 to 1.83 μg/L (Fig. S2).

### Sea urchin and HFO treatments

Adult sea urchins were provided from Dalian Haibao Fishery Co., Ltd. and transferred to the environmental toxicology laboratory. Animals were kept in tanks with circulating seawater until the exposure experiment was performed.

Sea urchins (horizontal test diameter: 52.5 ± 10.61 mm; vertical test diameters: 24 ± 5.66 mm) were randomly selected and placed in the treatment tank (20 sea urchins were raised in each tank). Two tanks (control and treatment) were controlled at 16 °C under a 12-h light: 12-h dark cycle for 21 d; three repeated experiments were arranged for each treatment. Sea urchins were fed kelp (laminaria japonica) every 3 d, and siphon was used to remove fecal waste from the bottom of each tank.

### Spawning, egg production and fertilization

After exposure, the sea urchin was washed with filtered seawater, and 1 mL KCl (0.5 M) was injected into the coelom through the peristomia membrane to obtain the gametes. To determine the spawning ability, the number of spawned male and female sea urchins was divided by the total number of male and female sea urchins according to Duan et al. (2018a). Each female’s reproductive ability was measured by the number of eggs that the female sea urchins excreted within 30 min (Rahman, Tsuyoshi & Rahman, 2002).

We used fertilization success as an indicator of sperm quality (Duan et al., 2018b). We used a pipette to collect 100 μL dried sperm in 10-mL filtered seawater, which was mixed quickly, then, the eggs released after 30 min were added and the beaker was gently shaken to fully mix the sperm and eggs. The fertilized eggs completely sank to the bottom of the beaker, then the shaking stopped. The seawater above the fertilized eggs was removed, then clean filtered seawater was injected, the fertilized eggs were washed, the process was repeated three times. After 15 min of fertilization, three duplicate samples (1 mL) were taken, few drops of 40% formaldehyde solution were added, and then, the fertilized eggs were observed using a microscope. Bulging of the fertilization membrane indicates successful fertilization (observed in at least 100 fertilized eggs per sample).

### Larval cultures

**Figure 1:**
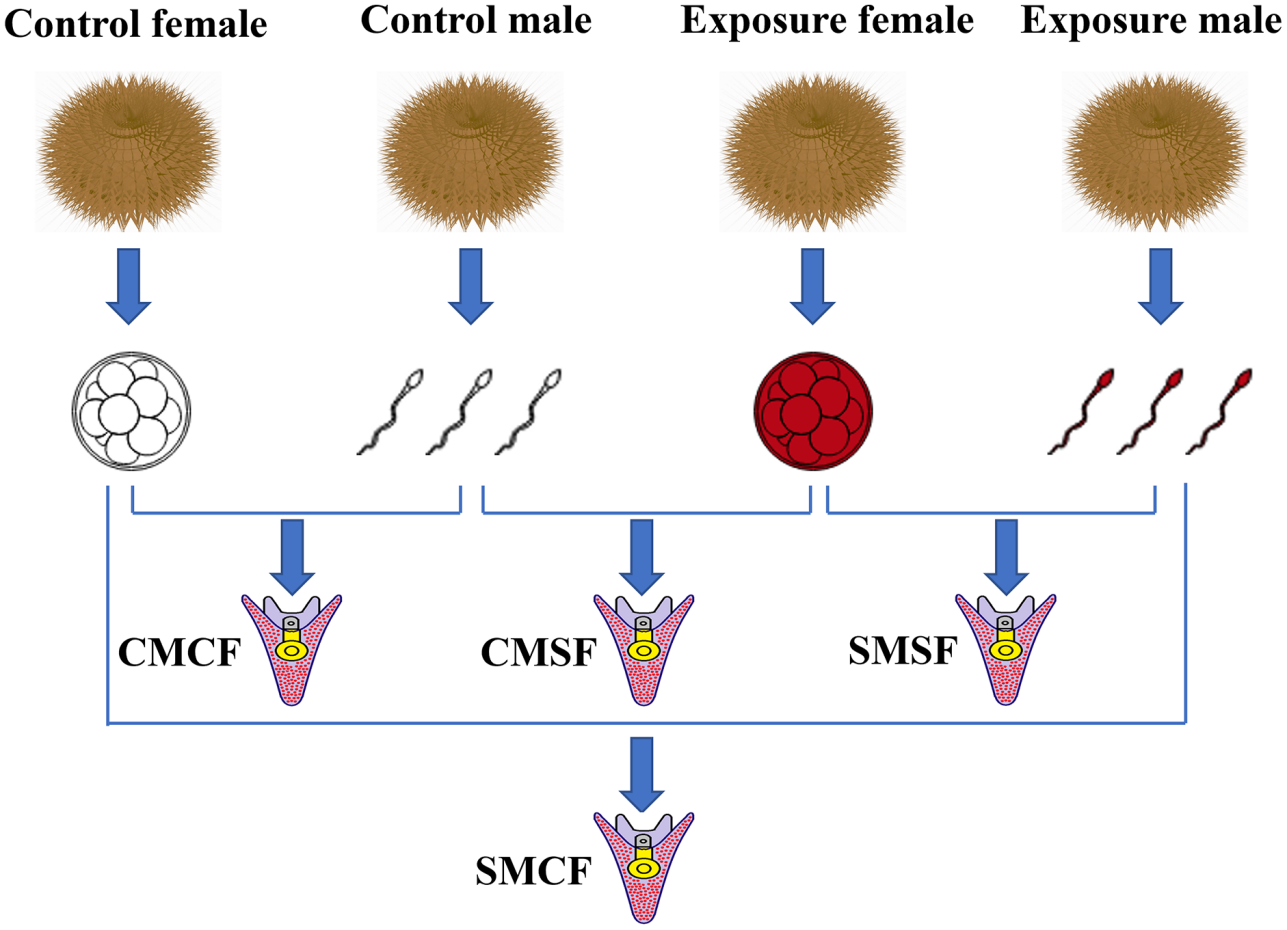
Schematic set-up of the experimental design. Sperm and eggs indicate that the gametes from either the control sea urchins or HFO exposure sea urchins. For the experimental design, there are four types of offspring: CMCF refers to control male crossed with control female, CMSF refers to control male crossed with exposed female, SMSF refers to exposed male crossed with exposed female, SMCF refers to exposed male crossed with control female.

Fertilized eggs were washed thrice to remove excess sperm. And then, they were cultured in buckets (diameter = 12 cm, height = 12 cm) with clean seawater and oxygen pumps, the embryos density was 200 ind/mL at 16 ± 0.5 °C. After 30 h, adjust the hatching density of fertilized eggs to 0.3–0.5 ind/mL. Subsequently, the larvae were fed microalgae (*Chaetoceros gracilis*) three times a day. The seawater (salinity 34 parts per thousand) was replaced two third each day using a fine silk net (Ding et al., 2020; Zhao et al., 2018). The embryos were produced with four types of parental crosses: CMCF (control male + control female), SMCF (exposed male + control female), CMSF (control male + exposed female), SMSF (exposed male + exposed female) (Fig. 1).

### Biological analysis

ROS production of sea urchin gonadal tissue was determined using a 2′,7′-dichlorodihydrofluorescein diacetate (DCFH-DA) method (LeBel, Ischiropoulos & Bondy, 1992) with the reactive oxygen species assay kit (Nanjing Jian Cheng Bioengineering Institute, Nanjing, China) according to the manufacturer’s described. Briefly, fresh sample (50 mg) was washed twice with 100 nM PBS buffer and then homogenized using homogenate instrument. The sample was centrifuged at 500*g* for 20 min and collected the precipitate. Then, the precipitate was mixed with 100 μL PBS and 50 μL DCFH-DA (1 mM) and incubated at 37 °C for 60 min. The fluorescence intensity was detected with excitation at 485 nm and emission at 525 nm using a Fluorescence spectrophotometer (Cary Eclipse; Agilent, Santa Clara, CA, USA). The ROS level was calculated in arbitrary units per mg protein (AU/mg protein).

3-NTP is an indicator of nitrosative stress and RNS production (Castellano et al., 2018). 3-NTP content in sea urchin gonadal tissues were measured using 3-Nitrotyrosine Elisa kit (Shanghai Yun Duo Biology, Shanghai, China). Brief, sample (50 mg) homogenate supernatants were mixed with standard and detection antibody horseradish peroxidase (HRP), and then washed thoroughly after incubation. The sample was developed with the substrate tetramethylbenzidine (TMB) and converted to yellow under the action of acid. The microplate reader ultraviolet spectrometry was used to measure the absorbance at 450 nm and calculate the sample concentration (nmol/L).

We used a protein carbonyl assay kit (Nanjing Jian Cheng Bioengineering Institute, Nanjing, China) to determine the total content of 2,4-Dinitrophenylhydrazine (DNPH) in the supernatant of sample (50 mg) homogenate (Reznick & Packer, 1994). The absorbance was measured at 370 nm using an ultraviolet spectrophotometer and calculated protein carbonyl content (nmol/mg protein).

### Gene expression of *hsp*70

Brief, the method of gene transcription was slightly modified based on Li et al. (2019). Total RNA was extracted from gonadal tissues and gametes and purified using the MiniBest Universal RNA Extraction Kit (Takara, Tokyo, Japan) according to the instructions. All samples were measured with an ultraviolet spectrophotometer to detect A_260_/A_280_ values, and the values of all samples were between 1.85 and 2.0, with an average RNA concentration of 721.6 ± 203.7 ng/μL. Total RNA integrity was evaluated using an agarose gel. cDNA synthesis of the sample was conducted using the PrimeScript™ RT reagent Kit with gDNA Eraser (Takara, Tokyo, Japan) and using MyGene™L Series Peltier Thermal cycler (LongGene, Hangzhou, China) at 40 °C for 15 min, and 80 °C for 5 s. Then, the LightCycler® Real-Time PCR System (Roche Diagnostics, Mannheim, Germany) was used to detect the expression levels of *hsp*70 and 18S (the reference gene) genes in the gonadal tissues and gametes, and the LightCycler® 96 Ver 1.1.0 Software was used for analysis. The conditions for a qPCR were: preincubation for 10 min at 95 °C, 3-step amplification (a denaturation step for 10 s at 95 °C, a hybridization step for 60 s at 60 °C, with an annealing step for 10 s at 72 °C) followed by 40 cycles. The 2^−ΔΔCt^ method was used to calculate the expression of *hsp*70 (Livak & Schmittgen, 2001). Primer sequences used in the study were listed in Table S1 (referred to Zhao et al., 2014).

### Larval size

**Figure 2:**
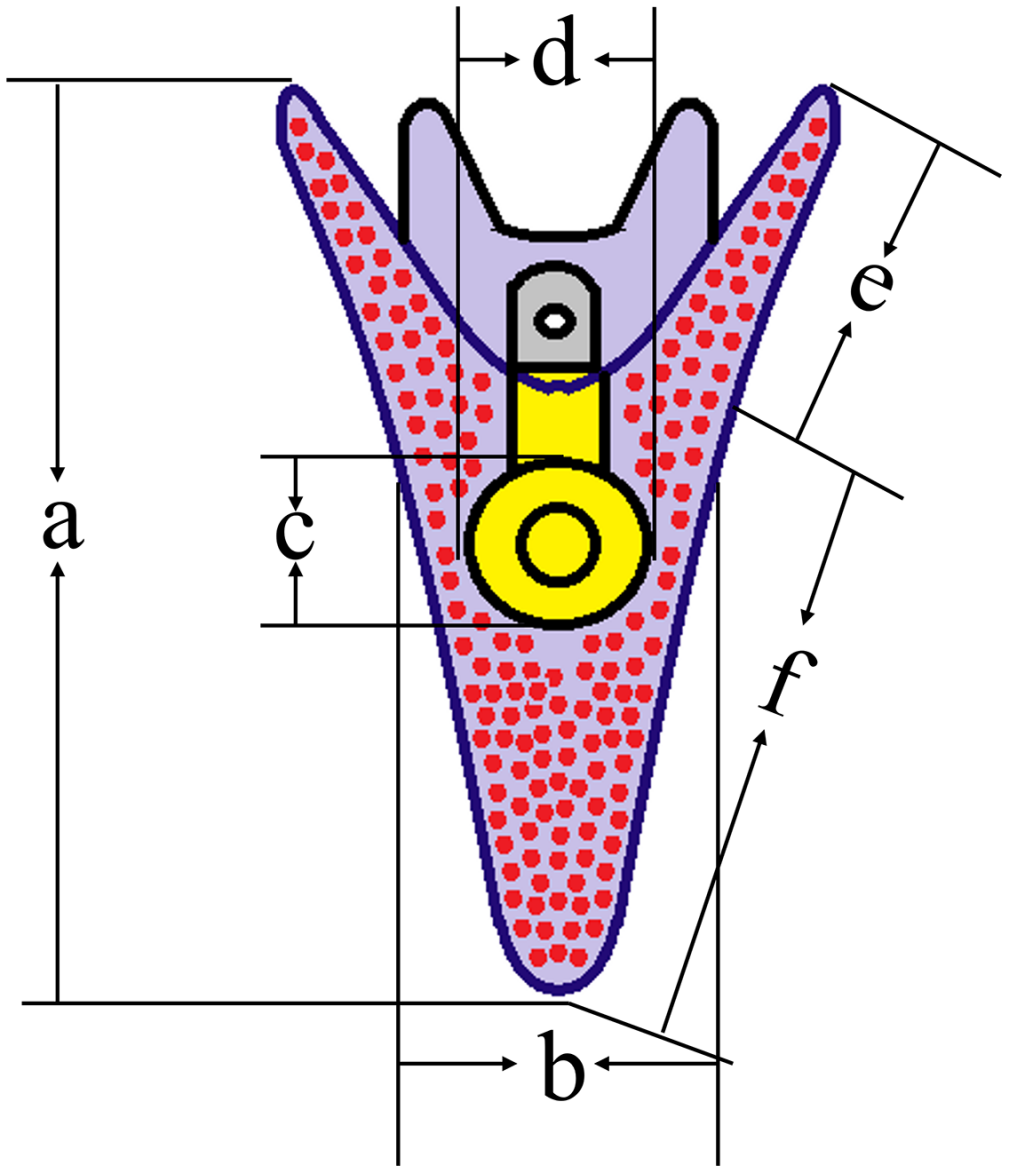
Conceptual diagrams showing 5 dpf larval size measurements. Lowercase letters a, b, c, d, e, f indicates larval length, larval width, stomach length, stomach width, post-oral arm length and body rod length, respectively. The size of normal larvae was measured as 446.88 ± 37.67 μm (a), 211.02 ± 34.99 μm (b), 78.36 ± 11.43 μm (c), 72.71 ± 8.45 μm (d), 94.65 ± 15.55 μm (e) and 352.23 ± 41.04 μm (f), respectively.

After 5 d post fertilization (5 dpf), we used a microscope (IX73; Olympus, Tokyo, Japan) to measure the growth-related traits of sea urchins (50 larvae for each group) (Zhao et al., 2018) (Fig. 2).

### Statistics analysis

The results were expressed as the mean ± standard deviation (SD) of the mean. One-way analysis of variance (ANOVA) followed by Tukey’s test were used for fecundity, *hsp*70 expression and larvae size. Spawning, ROS, 3-NTP and PC levels were analyzed by two-way ANOVA with sexes and treatments as fixed factors. It was considered statistically significant when *p* < 0.05. All results were analyzed using Sigma Plot 12.5 computer software (Systat Software, San Jose, CA, USA).

## Results

### Effect of HFO on spawning, egg production and fertilization

**Figure 3:**
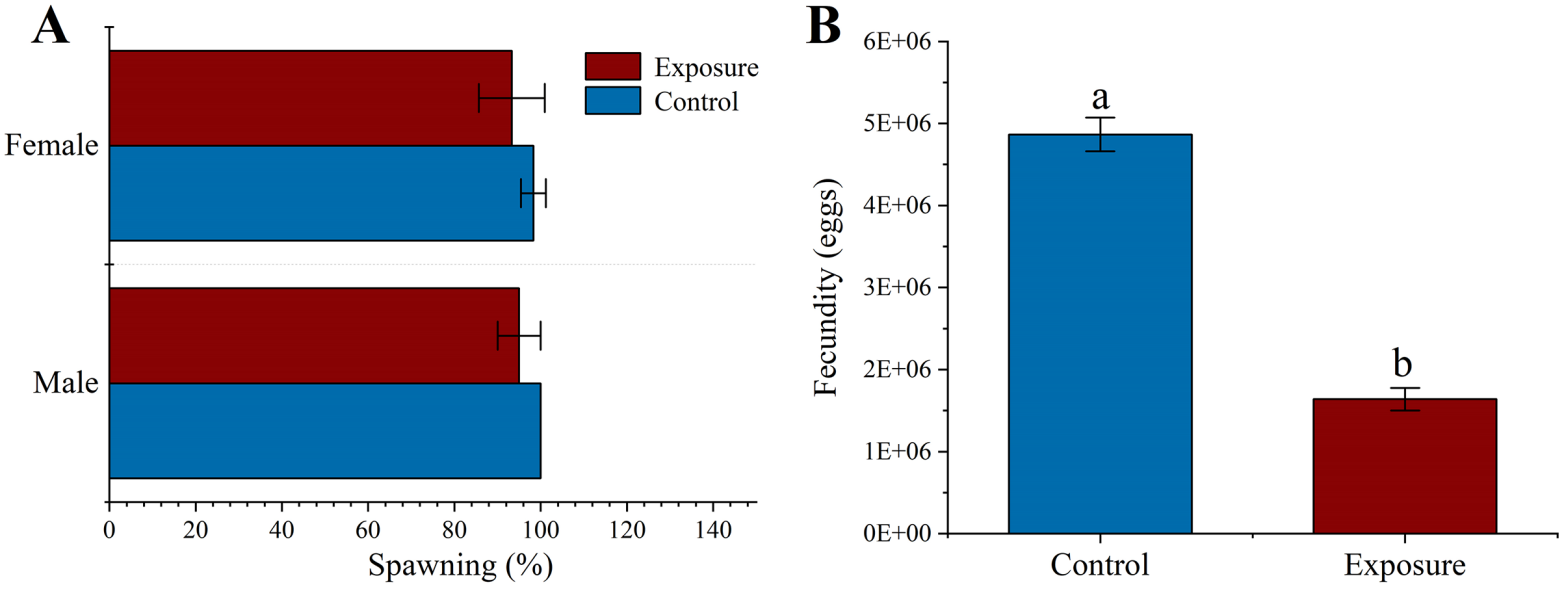
Effects of 21 d HFO exposure on gametes spawning (A) and fecundity (B) (mean ± SD). Different lowercase letters mean significant differences between treatments (*p* < 0.05).

There was no significant difference in spawning at the respective HFO concentration between different exposure treatments than control (*f* = 3, *p* = 0.158 and *f* = 0.125, *p* = 0.349 for male and female, respectively) or sexual groups (*f* = 0.1, *p* = 0.768 for exposure sexes) (Fig. 3A). Female sea urchins exposed to HFO had a significant decreased egg production (1.64 ± 0.14 × 10^7^ eggs) compared to control (4.87 ± 0.21 × 10^7^ eggs) (*f* = 507.06, *t* = 22.518, *p* < 0.001) (Fig. 3B). We observed the fertilization rate of each treatment, and the results showed that there was no significant difference between HFO treatment and control, and average fertilization rate of all treatments was 97.51 ± 1.73% (*f* = 0.43, *p* = 0.548).

### Effect of HFO on parental sea urchin

**Figure 4:**
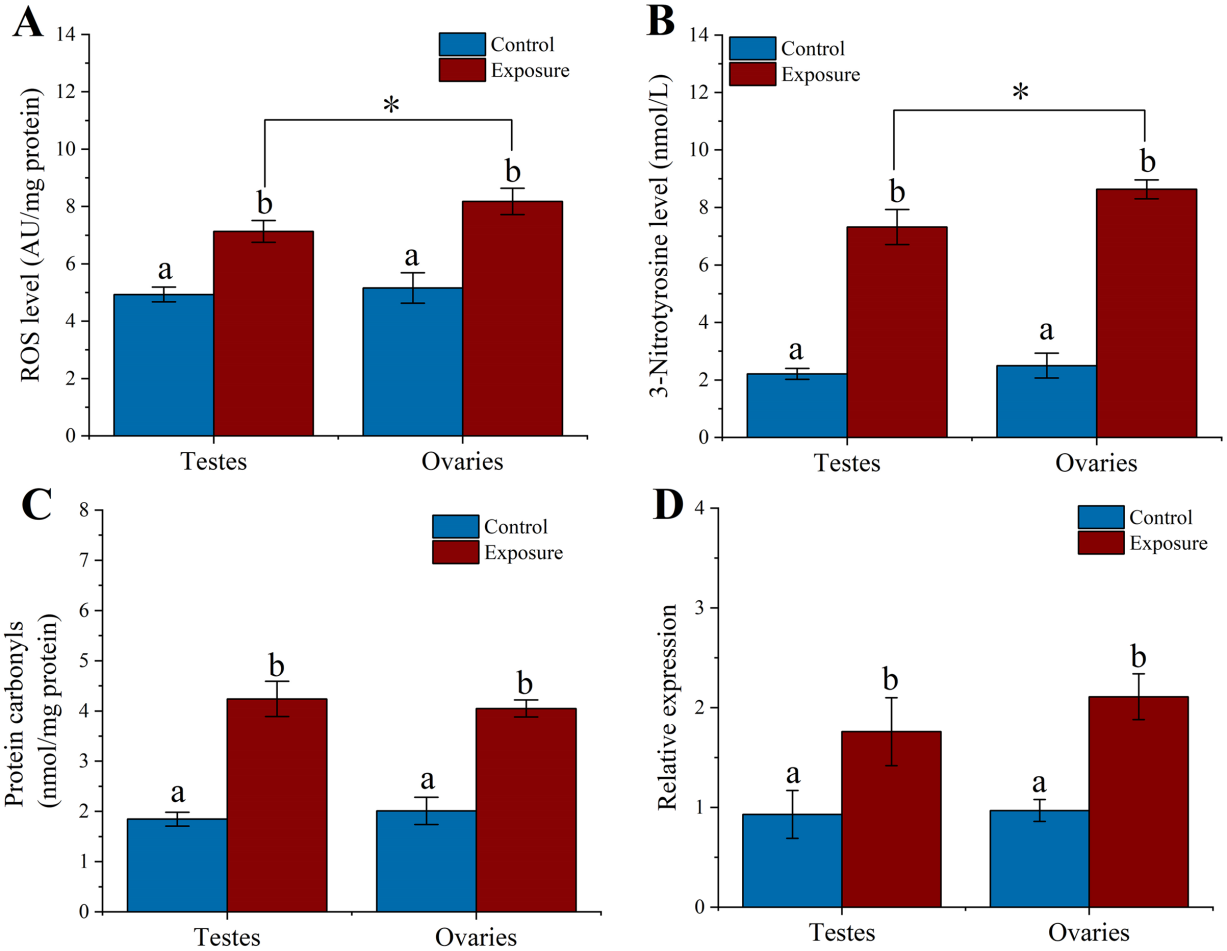
Effect of 21 d HFO exposure on ROS level (A), 3-Nitrotyrosine level (B), protein carbonyls level (C) and HSP70 expression (D) in sea urchin gonadal tissues (mean ± SD). Different lowercase letters mean significant differences between treatments (*p* < 0.05). An asterisk (*) indicates significant differences between testes and ovaries (*p* < 0.05).

To elucidate the excessive ROS production in sea urchin gonadal tissues caused by HFO, we analyzed the ROS level in sea urchin gonadal tissue after 21 d HFO exposure. Sea urchins exposed to HFO showed increases in ROS level in testes and ovaries, respectively. ROS level of testes and ovaries were significantly increased around 1.4- and 1.6-fold than control (*f* = 69.551, *t* = 8.34, *p* = 0.001 and *f* = 46.306, *t* = 6.805, *p* = 0.002, respectively) when sea urchin exposed to HFO. The ROS level of ovaries (8.18 ± 0.46 AU/mg protein) in sea urchin was significantly increased than testes (7.13 ± 0.31 AU/mg protein) after exposed to HFO (*f* = 10.86, *t* = 3.295, *p* = 0.03) (Fig. 4A).

3-Nitrotyrosine is considered as an indicator of apoptosis, cell damage, disease, and RNS production in aquatic organism (Curtis et al., 2011; Johnstone et al., 2019). Following 21 d of treatment, the level of 3-NTP in testes and ovaries of sea urchin was significantly increased by approximately 3.3- and 3.5-fold compared to control (*f* = 192.282, *t* = 13.867, *p* < 0.001 and *f* = 393.338, *t* = 19.833, *p* < 0.001, respectively) (Fig. 4B). The 3-NTP level of ovaries (8.63 ± 0.33 nmol/L) were significantly increased compared to testes (7.32 ± 0.61 nmol/L) (*f* = 10.655, *t* = 3.264, *p* = 0.031).

PC level were measured in gonadal tissues from two treatments. After 21 d treatment, we found that the PC level significantly upregulated ~2.3-fold in the testes (4.24 ± 0.35 nmol/mg protein) of sea urchins exposed to HFO compared to control (1.84 ± 0.14 nmol/mg protein) (*f* = 125.612, *t* = 11.208, *p* < 0.001). Meanwhile, PC level in the ovaries (4.05 ± 0.17 nmol/mg protein) showed significantly higher than control (2.01 ± 0.27 nmol/mg protein) (*f* = 126.807, *t* = 11.261, *p* < 0.001). However, there was no significant between testes and ovaries (*p* = 0.975) (Fig. 4C).

Heat shock protein has the function of preventing protein denaturation and recovering deformed protein. When activating the HSP genes, organisms can produce a class of specific proteins that repair and protect cells by rapidly regulating the cell defense system against oxidative stress (Wood, Brown & Youson, 1999). Sea urchins exposed to HFO showed an increase in *hsp*70 expression in testes and ovaries in male and female sea urchin, respectively. *hsp*70 levels in testes was significantly increased about 1.89-fold compared to control (*f* = 11.723, *t* = 3.424, *p* = 0.028). *hsp*70 levels in ovaries were also significantly increased 2.18-fold when female sea urchins exposed to HFO (*f* = 58.852, *t* = 7.672, *p* = 0.002) (Fig. 4D).

### Effects of HFO on gametes

**Figure 5:**
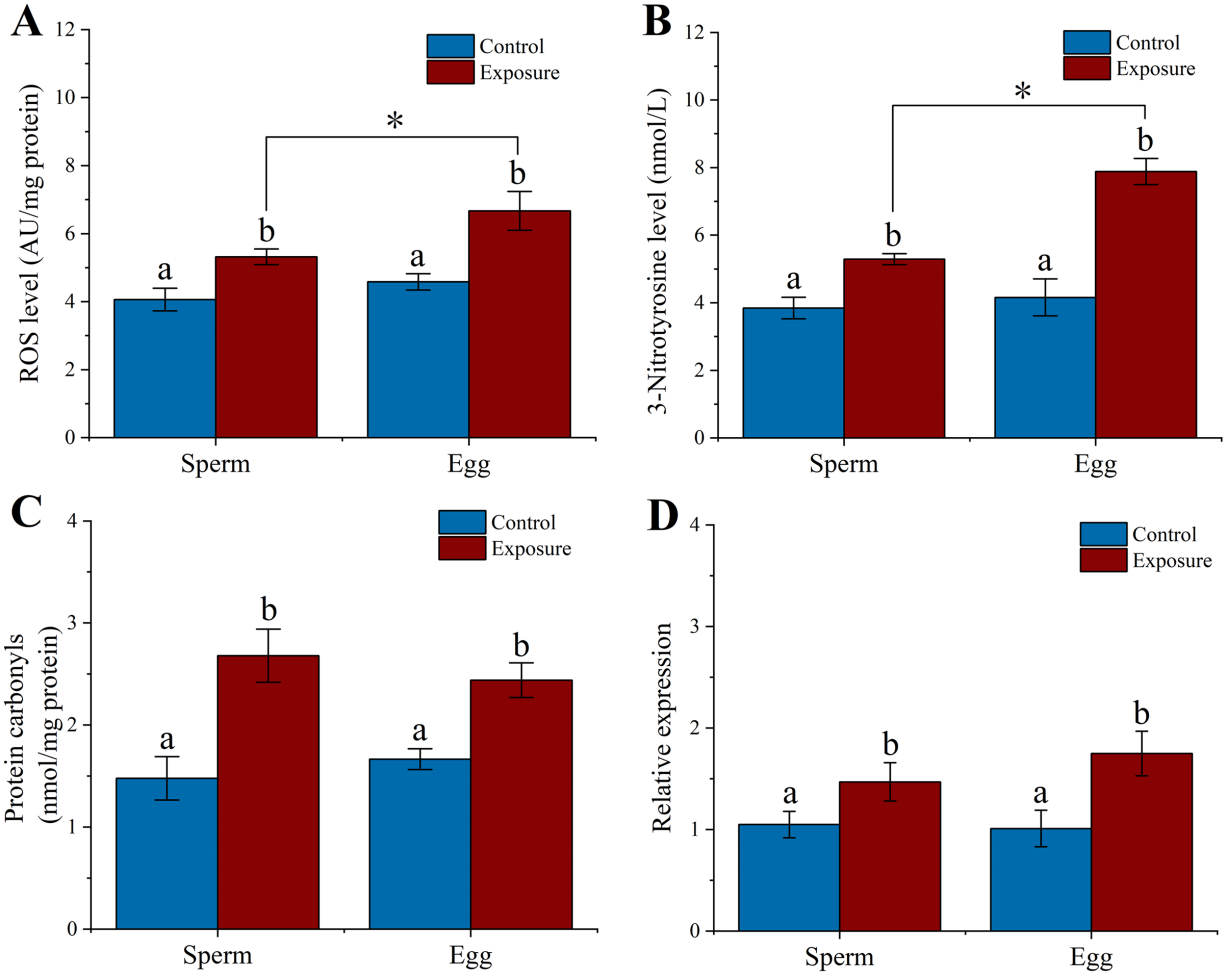
Effects of 21 d HFO exposure on ROS level (A), 3-Nitrotyrosine level (B), protein carbonyls level (C) and HSP70 expression (D) in sea urchin gametes (mean ± SD). Different lowercase letters mean significant differences between treatments (*p* < 0.05). An asterisk (*) indicates significant differences between sperm and eggs (*p* < 0.05).

We examined the level of ROS, 3-NTP, PC and *hsp*70 expression in gametes after sea urchins exposed to HFO for 21 d. We found that sperm (5.32 ± 0.23 AU/mg protein) and eggs (6.67 ± 0.57 AU/mg protein) released from exposure sea urchins showed significantly higher levels of ROS compared to control (control sperm: 4.06 ± 0.33 AU/mg protein, control egg: 4.58 ± 0.24 AU/mg protein) (*f* = 29.388, *t* = 5.421, *p* = 0.006 and *f* = 34.416, *t* = 5.866, *p* = 0.004, respectively). Eggs experienced a significant higher in levels of ROS compared to sperm following parental exposure (*f* = 14.35, *t* = 3.788, *p* = 0.019) (Fig. 5A).

Following 21 d treatment, the 3-NTP level in gametes (sperm: 5.29 ± 0.16 nmol/L, eggs: 7.88 ± 0.39 nmol/L) were significantly increased than control (control sperm: 3.84 ± 0.32 nmol/L, control egg: 4.46 ± 0.55 nmol/L) (*f* = 49.692, *t* = 7.049, *p* = 0.002 and *f* = 93.487, *t* = 9.669, *p* < 0.001, respectively). In addition, the level of 3-NTP was significantly higher in egg than in sperm following parental exposure (*f* = 116.075, *t* = 10.774, *p* < 0.001) (Fig. 5B).

PC level significantly increased 1.8- and 1.5-fold in the sperm and eggs when sea urchin exposed to HFO compared to control (*f* = 39.614, *t* = 6.294, *p* = 0.003 and *f* = 45.898, *t* = 6.775, *p* = 0.002, respectively) (Fig. 5C). In addition, we found that sea urchin exposed to HFO showed significantly increased in *hsp*70 expression in sperm (1.4-fold) and eggs (1.73-fold) compared to control, respectively (*f* = 9.975, *t* = 3.158, *p* = 0.034 and *f* = 21.025, *t* = 4.585, *p* = 0.01, respectively) (Fig. 5D).

### Larval size on 5 dpf

**Figure 6:**
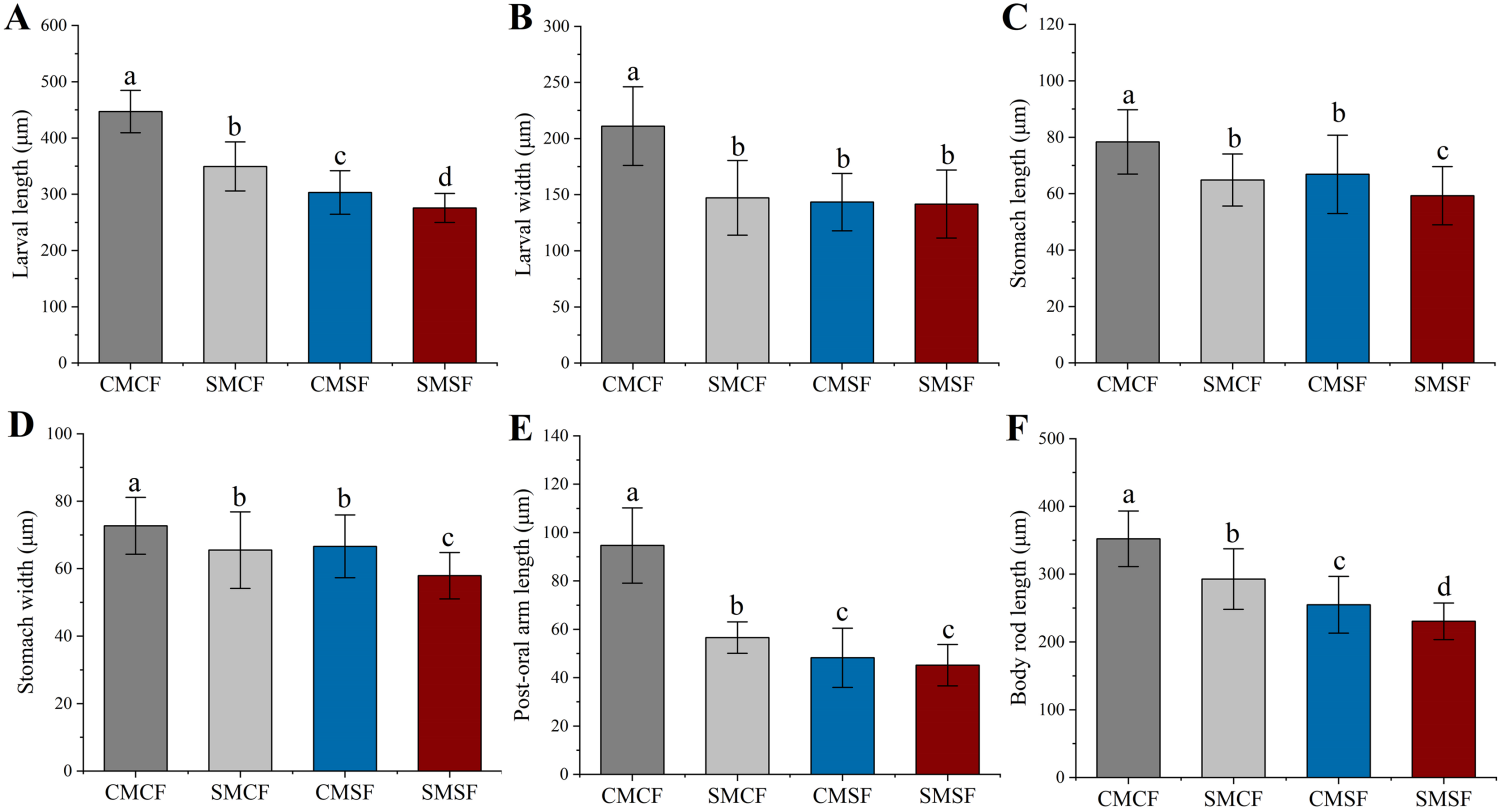
(A–F) Larval size of sea urchin on 5 dpf, whose parent exposed to long-term HFO or not (mean ± SD). The embryos were produced with four types of parental crosses: CMCF (control male + control female), SMCF (exposed male + control female), CMSF (control male + exposed female) and SMSF (exposed male + exposed female). Lowercase letters above the bars indicated significant difference among experimental groups (*p* < 0.05).

Parental sea urchin exposed to HFO have significantly negative effect on all larval size. SMCF were significantly longer in larval length, post oral arm length and body rod length compared to CMSF (*f* = 94.28, *t* = 9.71, *p* < 0.001 for larval length, *f* = 57.535, *t* = 7.585, *p* < 0.001 for body rod length and *f* = 54.443, *t* = 7.379, *p* < 0.001 for post oral arm length, respectively), but not for larval width (*f* = 1.238, *p* = 0.267), stomach length (*f* = 2.245, *p* = 0.135) and stomach width (*f* = 0.861, *p* = 0.354). In addition, SMCF were significantly higher in larval length (*f* = 318.792, *t* = 17.855, *p* < 0.001), stomach length (*f* = 24.34, *t* = 4.934, *p* < 0.001), stomach width (*f* = 49.14, *t* = 7.01, *p* < 0.001), post-oral arm length (*f* = 168.607, *t* = 12.985, *p* < 0.001) and body rod length (*f* = 214.405, *t* = 14.643, *p* < 0.001) than SMSF, not for larval width (*f* = 2.326, *p* = 0.128). Larval length (*f* = 52.482, *t* = 7.244, *p* < 0.001), stomach length (*f* = 29.09, *t* = 5.394, *p* < 0.001), stomach width (*f* = 84.836, *t* = 9.211, *p* < 0.001) and body rod length (*f* = 36.172, *t* = 7.244, *p* < 0.001) showed smaller size in SMSF than CMSF. However, the larval width (*f* = 0.306, *p* = 0.58) and post-oral arm length (*f* = 0.835, *p* = 0.125) of SMSF were not significantly different from those of CMSF (Fig. 6).

## Discussion

The results of this study clearly suggested that HFO exposure reduced the reproductive function of female sea urchins. In the study, the TPH concentration were decreasing from 811.22 to 474.64 μg/L, and PAHs concentrations decreased from 6.04 to 1.83 μg/L, within the concentration range after oil spill in the marine environment (Kim et al., 2010; Boehm, Neff & Page, 2007). The PAH distributions in HFO exposure solution was mainly phenanthrene and naphthalene. Egg production in female sea urchin showed a negative impact with HFO exposure, we found that the egg production in female sea urchins exposed to HFO decreased by 2.3-fold. A previous study showed that single PAHs exposure could prevented proper oogenesis and reduced fertility of sea urchin (Schaefer & Koehler, 2009). Organisms exposed to environmental stress exhibit increased degradation of ovarian follicles; a phenomenon referred to as follicular atresia (Santos et al., 2008). These data indicated that HFO exposure might inhibit egg formation in female sea urchins. In addition, we found that HFO has no effect on the spawning and fertilization of sea urchins, and the fertilization capacity after exposure could still reach more than 90%. However, a previous study found that the swimming speed of sea urchin sperm decreased after 8 weeks of exposure to oil production effluent (Krause, 1994). In this study, measurements limited to fertilization cannot clarify the harmful effects of HFO on the sperm quality of male sea urchins, which requires further investigation. The gonadal function would also be negatively affected by oil-derived hydrocarbons pollution in other marine invertebrates. For instance, the HFO contamination negatively affects the gonadal function of mussels (Bellas et al., 2008). Yang et al. (2021) found that scallop (*Chlamys farreri*) exposed to PAH reduced fertility. These results support that the HFO exposure decreases the gonadal function of sea urchins and other marine invertebrates.

ROS levels increase sharply in organisms under environmental stress, leading to oxidative stress, which in turn result in severe damage to their health and survival (Wang et al., 2019; Zhang et al., 2017). In addition, RNS are nitric oxide (NO) derivatives that contain highly oxidative free radicals and nitro compounds, which may lead to an increase in oxidative stress and apoptosis, thereby severely affecting the physiological state of organisms (Ahsan, 2013; Folkerts et al., 2001). NO reactions involve nitrification and nitrosation processes as well as RNS formation (Patel et al., 1999). Oil-derived hydrocarbons have been observed to induce ROS and RNS overproduction and oxidative damage in various marine vertebrate and invertebrate species. For example, Hong et al. (2016) found that populations of live Manila clam in oil spill damaged areas displayed high levels of ROS and RNS, that caused DNA damage. Donaghy et al. (2016) observed that mussels exposed to oil-derived hydrocarbons in seawater for 2 years after the 2007 *Hebei Spirit* oil spill had elevated levels of oxidative stress, and an altered energetic metabolism. In this study, we found that sea urchins exposed to stranded HFO showed higher levels of ROS, 3-NTP, and PC content in the gonadal tissue and gametes. This result indicated that the sea urchin gonadal tissues and gametes accumulated hydrocarbons-derived contaminants, leading to the overproduction of ROS and RNS and the oxidative damages, which may negatively impact sea urchin health.

Marine organisms should respond to the rapid changes in the aquatic environment and resist the environmental pollutants to maintain homeostasis for survival (Rivera Ingraham & Lignot, 2017). Heat shock proteins (HSP) are functional proteins in organisms that respond to the changes in the environment (Javid, MacAry & Lehner, 2007). *hsp*70 is an important member of the HSP family (Whitley, Goldberg & Jordan, 1999). The high expression level of *hsp*70 can protect and repair cells by rapidly adjusting the defense system, which assists in repairing protein damage under stress conditions (Clark & Peck, 2009). As a molecular chaperone, *hsp*70 can impede apoptosis by blocking the release of cytochrome C, Apaf-1 oligomerization, and caspase-8 activation. In addition, *hsp*70 has been recognized as a biomarker of environmental stress in adult sea urchin (Johnstone et al., 2019; Roccheri et al., 2004). *hsp*70 also excelled in reducing apoptosis and oxidative stress (Padmini & Tharani, 2014). In the stress response, *hsp*70 can regulate the cell defense system to resist stress and protect cells (Johnstone et al., 2019; Köhler et al., 1992). Our study showed that male and female sea urchins exposed to HFO experienced an increase in *hsp*70 expression in gonad and gametes. Madison, Hodson & Langlois (2015) observed that TPH of diluted bitumen (0.95 μg/L) significantly increase *hsp*70 expression in medaka embryos. Cruz-Rodriguez & Chu (2002) also found that suspended clay particles spiked with PAHs (400 μg/L) disrupted the metabolism of marine bivalve oysters, resulting in a significant increase in *hsp*70 level. These results suggested that HFO exposure increase the pressure on sea urchins, triggering more *hsp*70 expression to prevent contamination damage. Thus, *hsp*70 expression can serve as a biomarker for sea urchin exposure to different kind of pollutants, including HFO.

Despite the important gender confounding factors, few studies distinguished oil-related pollution sexual effects on sea urchins, and we found sex-specific differences in ROS and 3-NTP. We previously found higher polyunsaturated fatty acid contents in ovaries than in testes of sea urchins (Duan et al., 2018a). Kozhina, Terekhova & Svetashev (1978) found that eggs have a significantly higher level of polyunsaturated fatty acids contents than sperm. Our findings showed that ovaries and eggs had greater ROS and 3-NTP levels than testes and sperm after HFO exposure, respectively. Probably, in the presence of HFO derivatives, the higher energetic request for egg production in ovaries produces a high level of ROS and RNS with a higher oxidative potential for the oxidation targets such as polyunsaturated fatty acids, with possible cascade effect due to their modified structure.

Polyunsaturated fatty acids are substrates for ROS, RNS attack and production of lipid peroxidation (Lushchak, 2021). These results indicated that HFO exposure increased ROS and 3-NTP content in gonads and accelerated oxidative stress production. These processes resulted in gonadal oxidative damage and the transfer of hazardous sub-stances from parental individuals to their gametes, which may harm the growth and development of sea urchin offspring.

Phenotype of offspring may reflect that the petroleum hydrocarbon pollution influences the phenotypic plasticity and evolutionary adaptation of marine invertebrates. When parental sea urchins were exposed to stranded HFO for 21 d, the larval length of offspring whose parents were exposed to HFO were significantly shorter, and paternal exposure larval length were significantly higher than maternal and parental exposure. This may be due to paternal sea urchin exposed to PAHs had less oxidative damage on their early pre-feeding stages embryos (Lister, Lamare & Burritt, 2017). Our study revealed that the exposure of both parents to HFO resulted in a shorter larval stomach length and width. These results indicated a negative effect of HFO exposure on the stomach size of sea urchins. Larval post-oral arm and body rod lengths are related to the feeding ability and health of the sea urchins (Adams et al., 2011). After HFO exposure, the motor behavior of sea urchin larvae may be reduced, resulting in a weakening of the larval feeding ability. The energy obtained through feeding is distributed to cellular repair, reducing the energy supply for larval growth and development, thereby exhibiting degradation or delayed development of the post-oral arm and body rod (Zhao et al., 2016). Here, we found that the maternal exposure of sea urchins to HFO results in more harmful effects on the development of offspring.

## Conclusions

Oil spill is a great threat to marine invertebrates in shallow waters. Sea urchins live near the coast and are susceptible to contact with oil pollution due to their slow movements. In this study, we found that sea urchin exposed to HFO had a significant negative impact on the gonadal functions and gamete health, providing evidence for the parent-to-gametes transfer of stranded HFO toxic substances. The HFO exposure transmission effects are complex, and sea urchins exposed to HFO affect larvae size generationally. Paternal and maternal effects were highly trait dependent. We found that maternal individuals may play essential roles in the negatively effects of HFO exposure on larvae size. The results confirmed that female and male exposure to HFO can cause negative effects on their offspring which in turn affects sea urchin population maintenance and recruitment. This study could enrich our understanding of the persistent effects on the offspring of HFO exposure on sea urchins. The results of this study can provide relevant information for marine ecological risk assessment after oil spill accidents.

## Supplemental Information

10.7717/peerj.13298/supp-1 Supplemental Information 1 Raw data. Data on spawning, fecundity, fertilization, ROS level, 3-NTP level, PC level and larval size.

10.7717/peerj.13298/supp-2 Supplemental Information 2 The concentration of TPH in the solution of HFO during the 21 d exposure period.

10.7717/peerj.13298/supp-3 Supplemental Information 3 The concentration (A) and composition (B) of PAHs in the exposure solution.

10.7717/peerj.13298/supp-4 Supplemental Information 4 Data on the concentration of TPH in solution during exposure.

10.7717/peerj.13298/supp-5 Supplemental Information 5 Data on the concentration and composition of PAHs in the exposure solution.

10.7717/peerj.13298/supp-6 Supplemental Information 6 Primer sequences used for RT-qPCR.

10.7717/peerj.13298/supp-7 Supplemental Information 7 Raw data of HSP70 expression. Data on HSP70 expression in gonad tissues and gametes.

## Additional Information and Declarations

### Data Availability

The following information was supplied regarding data availability: The raw data are available in the Supplemental Files.
