# Supplementary figures and images for: Exposure of adult sea urchin Strongylocentrotus intermedius to stranded heavy fuel oil causes developmental toxicity on larval offspring
Source: PeerJ. 2022 Apr 19;10:e13298. doi: 10.7717/peerj.13298 (PMC9029359; doi:10.7717/peerj.13298)

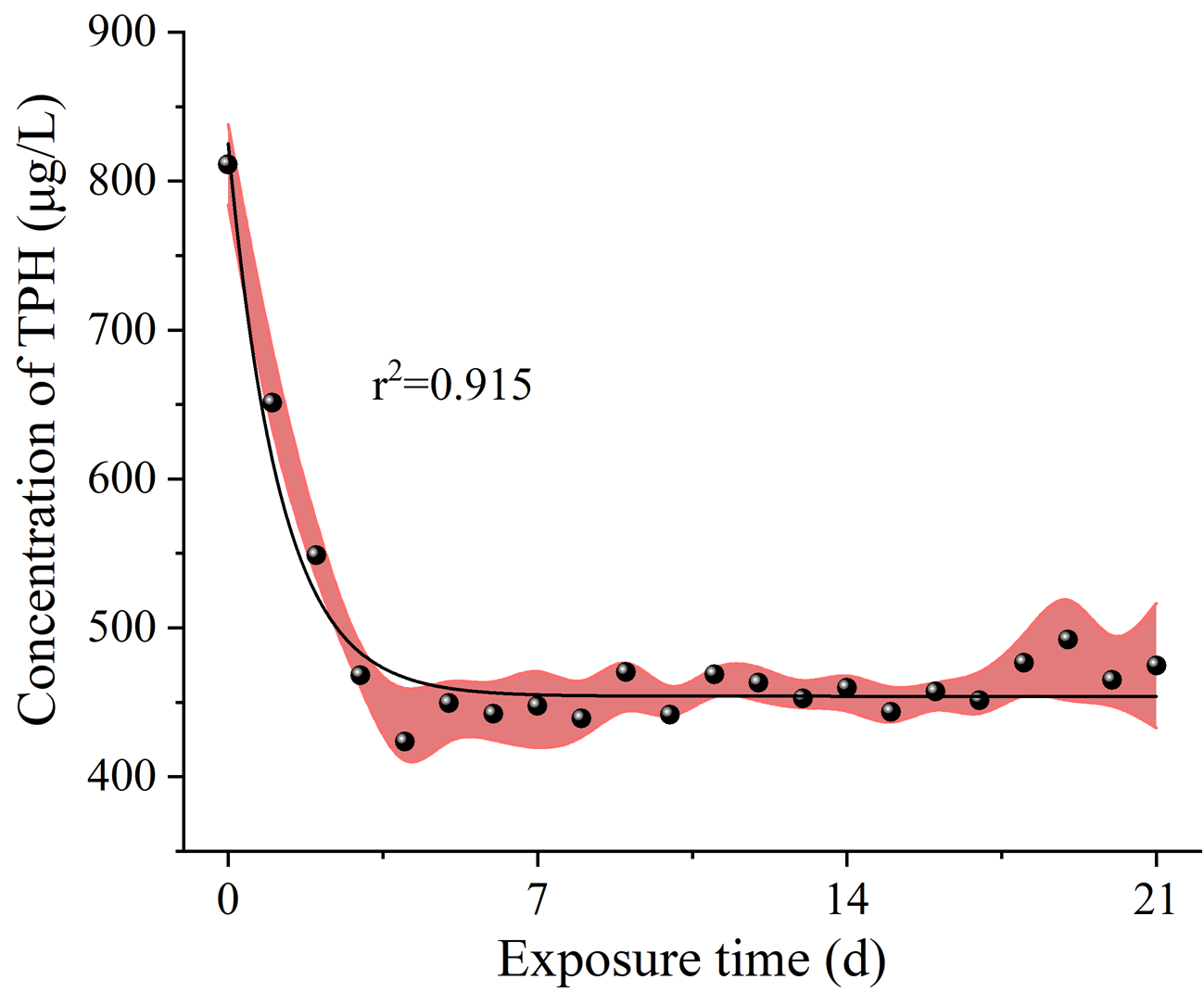

Supplement: Supplemental Information 2 [file peerj-10-13298-s002.png]

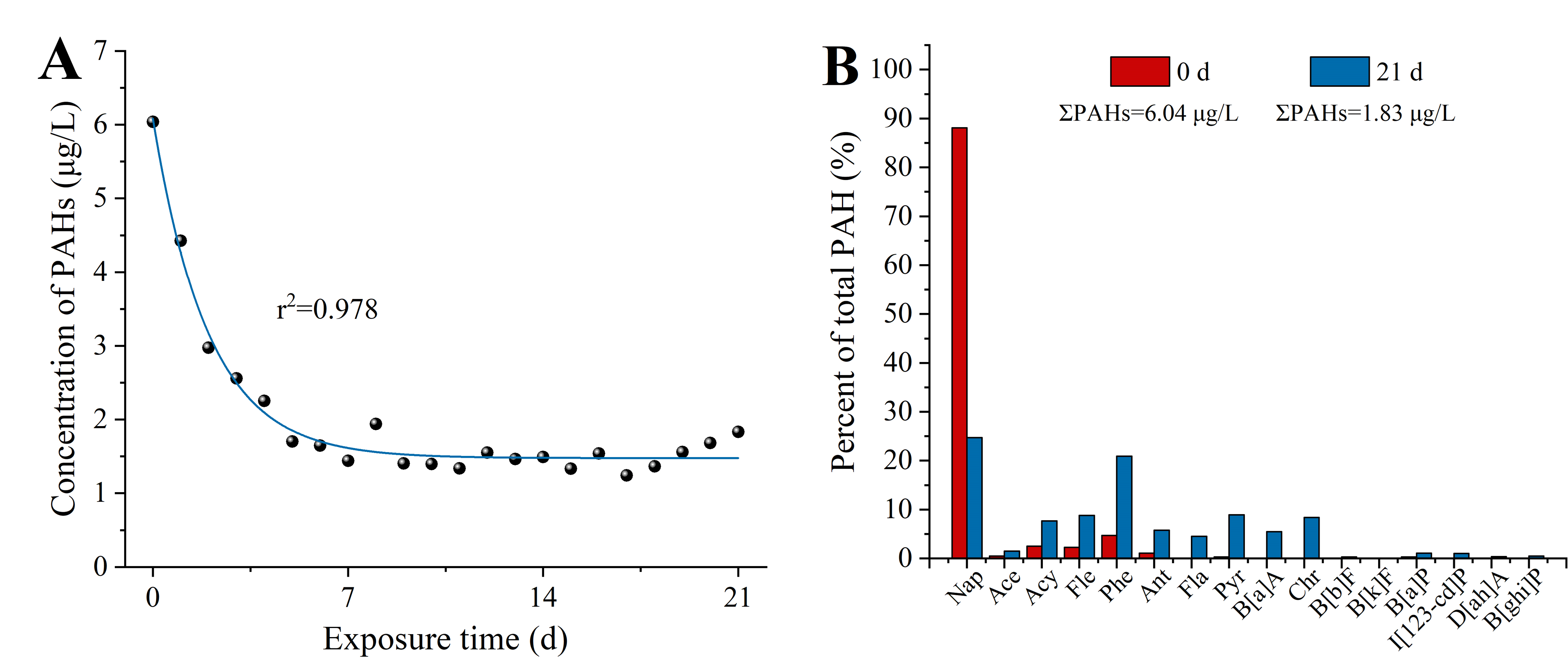

Supplement: Supplemental Information 3 [file peerj-10-13298-s003.png]
