# Supplementary material for: Exposure of adult sea urchin Strongylocentrotus intermedius to stranded heavy fuel oil causes developmental toxicity on larval offspring
Source: PeerJ. 2022 Apr 19;10:e13298. doi: 10.7717/peerj.13298 (PMC9029359; doi:10.7717/peerj.13298)
Supplement: Supplemental Information 6 [file peerj-10-13298-s006.docx]

**Table S1.** Primer sequences used for RT-qPCR.

| **Primer name** | **Prime sequences used for RT-qPCR (5’ to 3’)** | **Amplicon Length** | **Tm** |
| --- | --- | --- | --- |
| HSP70-F | ACACTCATCTCGGAGGAG | 144 bp | 54.95 |
| HSP70-R | CTTTCTTATGCTTTCGCTTGA |  | 54.88 |
| 18S-F | GTTCGAAGGCGATCAGATAC | 251 bp | 56.15 |
| 18S-R | CTGTCAATCCTCACTGTGTC |  | 55.80 |
